# What Shall We Do to Be Saved

**Published:** 1912-02-15

**Authors:** Wm. W. Belcher

**Affiliations:** Rochester, N. Y.


### WHAT SHALL WE DO TO BE SAVED.

WM. W. BELCHER, D.D.S., ROCHESTER, N. Y.

In this number we have an article on "An Objection to the Dental Nurse" in which the author says, "I have yet to hear the dental nurse advocated by any of the gentlemen who have had experience on the law committees of their District Dental Societies." If the gentleman will place his ear on the ground he will have his wish come true as I am about to make that statement.

Did I ever have any experience? To my sorrow, yes; I don't know it all, in fact the things I don't know about the subject would start a circulating library, but I know enough. When I think of the experience, it makes me feel like going out and slapping an orphan.

It was about ten years ago and I was ten years younger, that's one of the reasons why it happened. Things were bad, bad, not so bad as they are today but everybody agreed *someone* should do *something.* The Rochester Dental Society felt real grieved. As I was President, I became peevish about the matter.

We wanted to increase the membership and when we went to the younger men just starting in practice, they would say: "If this Dental Society of yours is so much, why don't you stop these illegal practitioners. Here's a fellow who didn't go but one term at my college, dropped out and went to work and here he is, as big as life, posing as the original Big Smoke, with good clothes and a Billiken Smile, a regular Busy Izzy and I with no business and no credit, holes in the bottom of my shoes and lace work around the bottom of my pants." Say Bo, do you call that a square deal? When you fellows get busy and arrest some of these people who are making our diplomas look like a magazine subscription coupon, we will join your society.."

This line of talk was a regular thing and you were made to feel as though you were inhabiting one of the condemned cells at Sing Sing. So, we wrote to the chairman of the State Law Committee and asked him to do something, but he was a cooney old boy and wasn't so easy smoked out of his hole and we wrote again and again; finally we received an answer in which the Chairman told us to get busy and secure evidence to convict and he would do the rest. Very quietly a few of the younger men got together and subscribed money to defray the cost of detectives fees and after a time we had evidence against six illegal practitioners, one of them a woman. We wrote the Chairman again with no response, wrote again and nothing doing. About this time we began to feel grieved and sent a red hot letter in which we reviewed the previous correspondence and said that we had gone ahead with childlike confidence, believing the seed we had sown was to come up as oyster plants with the shells on and pumpkins with the pies already baked but we were losing confidence; we had thought the Law Committee of the great State of New York a very fine piece of porcelain but the gilding was wearing away and we were loath to discover the common clay. This communication brought an answer. We were informed to keep ourselves in readiness and the learned attorney representing the Chairman would present himself in due time. They had their troubles in New York City and were loath to spend any money or time in a fresh water town the size of Rochester, but if we insisted they would attend to the matter with diligence. We thought our troubles were about ended and we began to feel like a real Peace Congress in full bloom.

The attorney came on from New York City; he was a man of broad experience and common sense. He reviewed our evidence and pronounced it fairly good; we had evidence of a few bonafide patients and the rest was of the hired detectives, this was of doubtful value, we were informed and we didn't feel so cheerful after spending our good money and cultivating cold chills and a perspiration in securing it.

A meeting of the Rochester Dental Society was called and the matter laid before them; we had worked in secret and few of the members up to this time knew anything about it. We were advised by our attorney to concentrate our energies on one case, the best one; secure a conviction in this case and afterwards, if we wanted to go on with the others, to do so, but he would advise having them sign a promise to discontinue practice and allow them to go their way unless they were caught again; in which event, the fact of their having been let off on a promise before the court to discontinue practice would make it comparatively easy to convict. Everybody was out for blood and a motion was passed with a grand whoop to prosecute every mother's son and the lady. We were assured that the work we had done for our society was the grandest ever and we were a noble band of heroes and were real worried for fear our Halo was not on straight.

The warrants were drawn up and then came the question, who was to sign them? The member of the Law Committee resided in a town a hundred miles away and anyway we had not called him in the fight as he wasn't well or for some other reason and it was up to *someone* to make the complaint and swear out the warrants. Guess who did it? Your Uncle Dudley. Everybody else was perfectly willing; someone had to do it and rather than have the performance stop, we acted as the Goat.

About this time we began to learn things that are not in the books. One of them was that the advice the lawyer had given us in regard to concentrating our efforts on one case was mighty good advice. When one man is arrested he feels as gay and chesty as a push cart peddler being chased off the corner by a blue coated cop; but we arrested them in bunches of six and they immediately formed a society among themselves for offense and defense. They commenced to pull wires, masonic, religious, social, political, friendship and relations were all worked to the limit, even the sunday school and pink tickets showing attendance played a part.

The opposing counsel was a very good lawyer and he had friends and well wishers all over the country. We went over jury lists to find the unacceptable ones, this man belonged to a labor union, this one was a "scab," another was a friend of the opposing counsel and this one was a friend of a friend. We served subpoenas on unwilling witnesses, gave up our time for a week, attending a post graduate course in dentistry, and the way the opposing counsel glared at us was almost an assault and battery. You were made to feel that you were in a nasty business and with half a dozen exceptions, the other members of the society refused to take any interest in police court affairs or to render any support. Some of the members who had been so red hot to arrest the whole half dozen and would not compromise, had a call from the men under arrest asking them how about the thing. They replied that they didn't have anything to do with it, they were rank outsiders; two or three were responsible for the whole affair.

Its a long street that don't end in a plowed field—even in Chicago—and it all came to an end. We convicted one man, or rather he pleaded guilty, one beat us out, two agreed to discontinue practice and the rest left town.

This experience made it very plain that it was as easy to convict a man of murder as proving illegal practice. You may be sure of his guilt but the next thing is to present evidence sufficient to convince "twelve good men, tried and true" with the smart lawyer to work on their sympathies and tell about this "poor boy with an invalid Mother to support" who we were *persecuting* and the only outcome would be to wreck his life and put the old lady in the poor house.

There never was a law made but it was violated, even that which says "Thou shalt not kill." So long as dentistry continues, will there be violations of the law. Today, we have more violators than ever in our city and they feel immune. Our efforts were of no benefit, it simply stirred up a lot of mud and no one, not even the public, was benefited.

### THE UNLICENSED GRADUATE.

Perhaps, there are, today, in the city of Rochester, half a dozen, yea, a dozen graduates of colleges outside of New York State who have not passed the examining board and never will; they have no high school education and cannot make up their counts to take the examination. There are good dentists among them too. Now, do you think if they were arrested and brought up before a jury, there would be a chance of their conviction? The intelligent jury would ask, "What's the matter with this man? Isn't he a graduate?" "He is." "The counsel for the defense tells us he is a graduate of the University of——— Dental Dept. Isn't that a good dental school?" "One of the best." "Why then are you persecuting this young man if he is a graduate of an excellent college?" "He isn't a graduate of a high school," you reply. "What difference does that make if he is a graduate of one of the best dental colleges in the country and the testimony has shown he is a competent workman, nay, you acknowledge this yourself." Do you suppose any jury is going to convict in this case? You can arrest and rearrest and so make it unprofitable for him to continue but meanwhile you have to live and he can start practice in another and a greener field.

A high school or a dental college does not necessarily make a man a better dentist, but all other things being equal this preliminary preparation is of the greatest value and there never was a law but worked hardships on a few. With my experience, I would gladly see every law on the statute book regulating the practice of dentistry thrown to the winds. Then every tub would stand on its own bottom and the public would begin to inquire in regard to a man's preparation and if he was a graduate and from what school. As it is now, they take it for granted he is qualified to practice as they know there are laws enacted for their protection, and if a man holds himself out to practice, he must be O. K. or he would not be allowed to continue.

This brings us to the point of asking: "Why were these laws enacted?" For the protection of a few dentists? No, sir. They were enacted for the PROTECTION of the public that they would be safe from the unskillfull and the charlatan. Incidentally, the dental profession profits by these laws, or they think they profit. I do not believe it is so.

The fact remains that the dental profession has as good laws as we deserve and they are enforced with the diligence deserved. The mass of the profession are rank cowards when it comes to enforcing their laws; they havn't the backbone of a jelly fish and they are perfectly willing someone else make the sacrifices and when it comes to taking any part in the enforcement of their laws, they fade away like a phantom of the desert. Their great concern is that they shall not be known or appear in any way for fear they make enemies or lose practice. Men will write in from country towns telling you of their neighbor and his doings, but "Don't mention my name, for God's sake, it would hurt my business and this man has influential relatives who would resent my mixing up with his affairs." Isn't that noble and grand?

SUPPORT OF THE PUBLIC.

No law can be enforced without it have public opinion back of it and this the dental law has not. It is thought, even among intelligent people, that the dental laws are for the protection of the dental profession who have a big trade union and the laws help them to keep up fees and exploit the public. This is part of the advertising man's stock in trade and he uses it to his advantage.

And with this experience you favor the employment of a dental nurse? I do. She couldn't be half as bad as her male confreres and at the present time with ten to twenty percent. of illegal practitioners in the field, things could not be much worse. Then the woman lacks the initiative and cares more for public opinion. Personally, I do not feel that she would ever become a factor in illegal practice.

As the man from Battle Creek says: "There's a Reason." Also let it be said, there's a remedy. First of all you must have the public back of any movement for the suppression of the illegal practitioner; they must be as much interested as you and be made to understand the law is primarily for their protection and benefit. This point has been demonstrated in an action brought by the State Dental Society of Illinois against a dentist for unprofessional conduct and revocation of license, (the crime in this case was that of assaulting a female in his office) the court held that unprofessional conduct in the eyes of the law was not that of a violation of the code of ethics but rather a violation of the dentist in his relation to the public. For instance, a dentist holding out to perform dental operations without pain, is guilty of unprofessional conduct, as he is promising something he cannot fulfill; in other words he is deceiving the public for his private gain.

Here is a plan to clear your vicinity of the illegal practitioner and if it is carried out in its entirety it cannot help but be successful. It requires faith and departing from the accepted practice, but the methods of the past have shown themselves as absolutely inefficient and only serving to create bad feeling among the general public and a burden of unmerited reproach to the ones most evident in any attempt to cleanse a dirty stream by beginning at the end rather than its source.

Obtain evidence against the most flagrant violator of the law of not only unprofessional practice but conduct, and several months before the meeting of your State Society, swear out an affidavit against this dentist, and present this evidence of unprofessional conduct to the Board of Censors and ask that his diploma be revoked. The dentist will have an opportunity at a date set by the Board to refute charges. Also secure evidence against a man who is violating the law in practicing without registration.

### CALL A MEETING.

As early as possible and before the meeting of the State Society, send out a general call for a meeting of the District Society and invite EVERYONE to be present, make it an open meeting in which every dentist, no matter what his standing, will be asked to join. Have present the best speakers and men of the profession that can be secured, have a banquet and music; present the facts of an affidavit having been made against a dentist for unprofessional conduct, also of illegal practice; make it plain that the District is to be cleansed of illegal practitioners, ask the co-operation of all present and invite them to join in the work. State that from this time on, no one shall practice illegally and the dentists who have had charges brought against them are only the beginning, and all who violate the law will be similarly dealt with. See that the public press is is in touch with the full details of the affair and that this is for the benefit of the public and not the dental profession and all men of reputable practice are behind the movement. That all are equally concerned and each man ready to do his part and take the responsibility. We would no longer find it necessary to wash our dirty linen before the public. Every man who so desired would have an opportunity to take part in this meeting and become a part of the working force without prejudice as to his former actions or standing. In case of further violations, make out affidavits against these men and have some friend of his inform him he is next on the list, that he had his chance to join the society and act and conduct himself with decorum or get out of the District as he would be made a show of, his case presented to the State Society for settlement and his diploma taken away from him. With this plan and the co-operation of the press and the public the conditions can be made ideal. The public will not stand for any close corporation and must be informed of each step and their help and support secured. Like most things worth while it would take some effort and money. If we do not want to do this, then the profession must continue in its hog wallow and make the best of it.—*The Dispensary Record.*

